# Effects of Dribbling constraints on Sprint Acceleration Performance and the Force-Velocity Profile according to Playing Positions in Professional Soccer Players

**DOI:** 10.5114/jhk/204820

**Published:** 2025-11-20

**Authors:** Dhia Benhassen, Abderrahmane Rahmani, Firas Zghal, Pierre Samozino, Haithem Rebai, Nicolas Peyrot

**Affiliations:** 1 Movement - Interactions – Performance (MIP), Le Mans Université, Le Mans, France.; 2 Research Laboratory Education, Motricité, Sport et Santé (EM2S), University of Sfax, Sfax, Tunisia.; 3 Complexity, Innovations, Motor and Sports Activities (cIAMS), Université Paris-Saclay, Gif-sur-Yvette, France.; 4 Complexity, Innovations, Motor and Sports Activities (cIAMS), Université d’Orléans, Orléans, France.; 5 Inter-University Laboratory of Human Movement Sciences, University Savoie Mont Blanc (LIBM), chambery, France.; 6 Research Laboratory Sport Performance optimization, National center of Medicine and Science in Sports, Tunis, Tunisia.

**Keywords:** power, sprinting, acceleration, playing position, dribbling

## Abstract

This study aimed to investigate the impact of dribbling on sprint acceleration performance (sprint time) and the associated force-velocity (F-V) profile among professional soccer players across playing positions. Participants (N = 52) were categorized as central defenders (cDs), wide defenders (WDs), central midfielders (cMs), wide midfielders (WMs), and forwards (Fs). A field method based on split times measurements during 30-m sprint acceleration was used to calculate maximal theoretical force (F_0_) and velocity (V_0_), maximal power (P_max_) and the F-V slope during sprinting without the ball and while dribbling. Our study revealed a significant decline in sprint performance during dribbling. cDs and WDs exhibited higher losses in 5-m split time (T5; 19%) compared to Fs (13%) and WMs (14%). cMs displayed higher losses in 20-m split time (T20; 11%) compared to Fs (8%). P_max_ and F_0_ significantly decreased when dribbling, with a higher decrease in P_max_ experienced by cMs (33%) compared to Fs (24%) and WMs (25%). Similarly, a higher decrease was observed in F_0_ for cMs (29%) compared to Fs (20%) and WMs (20%). The determination coefficients between the loss in T5 and P_max_ (r^2^ = 0.54), F_0_ (r^2^ = 0.62), and the loss in T20 and P_max_ (r^2^ = 0.75), F_0_ (r^2^ = 0.23), and V_0_ (r^2^ = 0.22) indicated substantial relationships between sprint performance decline and losses in the F-V profile. In conclusion, this study highlights how dribbling affects sprint performance and the F-V profile differently across soccer playing positions. coaches can tailor training programs considering the constraints imposed by dribbling for effective players’ development.

## Introduction

Sprint accelerations are crucial actions for achieving success in soccer (Faude et al., 2012), leading to goal-scoring or playing a pivotal role in creating goal-scoring opportunities (Sweeting et al., 2017). These decisive actions often require player's ability to sprint while dribbling, a hallmark trait of gifted soccer players (Bekris et al., 2018), which decisively shapes the game outcome (Huijgen et al., 2014). However, a recent investigation in U16 soccer players showed a 28% decline in linear sprint performance while dribbling (Manouras et al., 2023). While it is essential to closely monitor the decline in linear sprint performance during dribbling, it is also important to consider the influence of different playing positions. Previous studies have indicated that players in different positions display varied sprint activity and high-intensity running patterns during match play and training sessions (Asian-clemente et al., 2024; carling, 2010; Di Salvo et al., 2007), with offensive positions showing faster linear split times compared to other playing positions (Hassen et al., 2024; Haugen et al., 2013). However, there is a lack of studies examining differences in sprint performance during straight- line dribbling versus classic sprint tests, especially across playing positions.

To better understand the mechanical determinants of sprint acceleration performance, horizontal force production capacities during classic sprints among soccer players have been well studied by assessing the sprinting force-velocity (*F-V*) relationship. Indeed, horizontal force production capacities during sprinting at high and low velocities are two distinct qualities with high interindividual variability, leading to very different *F-V* profiles among athletes, even when they present similar acceleration performances (Hassen et al., 2024; Haugen et al., 2020). Exploring the sprinting *F-V* profiles has enabled a better understanding in various aspects, such as players' levels and capacities (Devismes et al., 2021; Jiménez-Reyes et al., 2018), performance evaluation and optimization (Morin and Samozino, 2016), and training guidance (Morin and Samozino, 2016). Notably, exploring the sprinting *F-V* profile has significantly contributed to a better understanding of acceleration performance determinants in soccer players (Devismes et al., 2021; Hassen et al., 2024; Marcote Pequeño et al., 2018). Interestingly, it has been shown that the sprinting *F-V* profile differs according to playing positions, with forwards exhibiting the highest horizontal force at high and low velocities (*V_0_* and *F_0_*_,_ respectively), leading to highest maximal power output (*P_max_*) and thus, improved sprint acceleration performance (Hassen et al., 2024; Haugen et al., 2013, 2020). Position-specific technical and tactical demands likely contribute to variations in the *F-V* profile, as sprinting distances and occurrences differ across playing positions. Additionally, recent studies on acceleration-speed profiles, derived from GPS data collected during training sessions, have further advanced our understanding of soccer-specific performance. These profiles capture the relationship between maximal acceleration and top speed, providing a practical tool for assessing sprint performance (Morin et al., 2021). Notably, acceleration-speed profiles are particularly valuable for analyzing in-game movements, as they highlight variations in acceleration and speed capacities that are closely linked to players' positional roles and the tactical demands of the game (Alonso et al., 2022). Such variability in these profiles underscores the importance of aligning players’ selection and training programs with specific positional requirements, enabling coaches to optimize physical capacities in line with tactical roles.

Previous studies have primarily focused on the sprinting *F-V* profile under optimal conditions (Devismes et al., 2021; Hassen et al., 2024; Samozino et al., 2022), without considering the technical constraints associated with sprinting while dribbling with the ball (Huijgen et al., 2014). Indeed, sprinting when dribbling is a complex task that involves a combination of technical skills, coordination, and physical abilities (Sheppard and Young, 2006). Sprint acceleration performance when dribbling is thus likely decreased compared to maximal sprint capacities due to the decline in the ability to produce maximal propulsive power (*P_max_*), itself resulting from the alteration in the ability to produce horizontal force at high and/or low velocities (*V_0_* and *F_0_*_,_ respectively). Moreover, variations in technical and physical capabilities among playing positions can greatly influence sprint performance when dribbling (Dellal et al., 2011). Previous reports suggest that forwards exhibit more frequent sprint actions while dribbling during match play compared to other positions (Oliva-Lozano et al., 2023), potentially resulting in a more notable preservation of horizontal force production capacity. To delve into the intricacies of sprint acceleration while dribbling, it is crucial to consider the potential variability in the ability to produce horizontal force at high and low velocity, depending on playing positions. The sprinting *F-V* profile emerges as a valuable tool for dissecting the nuances of this skill, investigating the ability to accelerate while dribbling, distinguishing between low and high velocity production during dribbling sprints. Such an approach enables a detailed assessment of players' performance in dribbling situations across playing positions, thereby identifying key factors (e.g., biomechanical factors) influencing in-match sprint acceleration and game outcomes.

Therefore, the main aim of this study was to compare the effects of dribbling on sprint acceleration performance among professional senior soccer players, depending on their playing positions. We hypothesized that the decrease in sprint acceleration performance when dribbling, compared to sprints without the ball, would be lower for forwards compared to other positions. The second aim was to assess the effects of dribbling on the sprint *F-V* profile among soccer players across different playing positions. We expected distinct alterations in *F-V* profile variables, particularly *F_0_* and *V_0_*, during dribbling compared to classic sprints, with potential variations between forwards and players in other positions.

## Methods

### Participants

A total of fifty-two (n = 52) professional senior soccer players belonging to three Tunisian first division clubs participated in the study (age: 25.10 ± 3.4 years; body height: 179 ± 0.07 cm; body mass: 75.1 ± 7.7 kg). All participants were outfield players categorized into five groups by their playing position: central defenders (cDs, n = 10), wide defenders (WDs, n = 10), central midfielders (cMs, n = 12), wide midfielders (WMs, n = 11), and forwards (Fs, n = 9). All players followed a six-day-per-week in-season soccer training program, including matches, with sessions focusing on technical, tactical, and overall physical ability improvement. Participants had no lower limb injuries six months prior to the study and were medication-free during the research. Players who joined the team after the resumption of training in July 2022 were excluded. All players provided written consent following ethical guidelines, and the study was approved by the local ethics committee from the High Institute of Nursing, University of Sfax, Sfax, Tunisia (protocol code: cPP/SUD N0367/2021; approval date: 23 November 2021), aligning with the Declaration of Helsinki.

### Measures

The experimental protocol was conducted during the in-season period to ensure the integrity of *F-V* profile determinants, avoiding pre-season and end-of-season fluctuations. Assessments occurred consistently in November–December 2022. Each player completed two trials under both testing conditions (a sprint without the ball and a dribbling sprint), with only the best sprint performance from each condition used for the *F-V* profile analysis. All players completed the assigned trials under both conditions without any withdrawals or missing data.

Players were asked to avoid strenuous activity for 48 h before testing. Initial anthropometric measurements, including body mass (BM, kg) and the stature (H, cm), were recorded. Testing was preceded by a standardized 20-min warm-up routine consisting of jogging, dynamic stretching, sprints, and ball exercises, followed by a 3-min passive recovery period to clarify the procedures. One week before testing, players were familiarized with the protocol on a grass-surfaced playing field. Testing sessions were conducted on the same grass surface, with players attired in usual training gear.

### Design and Procedures

A cross-sectional design was used to assess horizontal force production capacities during sprinting in professional soccer players, comparing sprinting without the ball with dribbling sprinting.

An a priori sample size estimate was calculated using G*Power (Version 3.1.9.4) to determine the number of participants necessary to detect differences in key sprint variables between groups with and without dribbling. Based on a previous study (Silva et al., 2022), a minimum sample size of 35 participants was determined to be necessary to achieve adequate statistical power for our study using a two-way ANOVA (power = 95%, α = 0.05, 5 groups).

### Sprints Testing Procedures

Following the warm-up, athletes performed two 30-m classic linear sprints and two 30-m linear sprints while dribbling, with a 3-min rest interval in between. All sprints began from a staggered-stance position with the right hand on the track. Dribbling sprints followed the protocol outlined by Gouvêa et al. (2017), requiring a minimum of four ball touches during sprinting. Additionally, the player must have maintained control of the ball and crossed the finish line with the ball at their feet. No starting signal was provided, allowing athletes to initiate the sprints at their own discretion.

All trials were recorded using an iPad device (iPad 11 Pro; Apple) equipped with a high-speed camera (240 fps, 1080p quality), positioned frontally on a 10-m tripod perpendicularly to the sprint direction, just in front of the 15-m marker. Sprint initiation was defined as the moment when the athlete initiated a forward movement in their lower limbs. Video analysis via the My-Sprint app (Romero-Franco et al., 2017) provided split times (at 5, 10, 15, 20, 25 and 30 m) for each trial. In our study, we assessed the reliability of the trials for both sprint without the ball and dribbling sprint tests based on intraclass correlation coefficients (Iccs) and standard error of measurements (SEM). For sprint without the ball tests, the Icc values exceeded 0.90 (95% cI: 0.80–0.97), and the SEM averaged 1.02% ± 0.11% for sprint performance. In terms of sprint *F-V* variables (*F0, V0*, and *P_max_*), the Icc surpassed 0.80 (95% cI: 0.59–0.95), with the SEM averaging 3.13% ± 0.74%. Similarly, for dribbling sprint tests, the Icc values exceeded 0.83 (95% cI: 0.65–0.94), with the SEM averaging 1.42% ± 0.25% for sprint performance. However, for dribbling *F-V* variables, the Icc surpassed 0.63 (95% cI: 0.26–0.92), and the SEM averaged 4.8% ± 1.24%. The R^2^ values for the *F-V* relationship model were consistently equal to or greater than 0.99 in all analyses, indicating a very strong fit of the model to the data. This highlights the model’s robust performance and the reliability of our analysis and results for both sprinting without the ball and dribbling sprint tests.

According to the method proposed by Samozino and colleagues (2016), split times from the best attempt (30-m time), along with anthropometric variables [“H” (cm) and “BM” (kg)] and meteorological conditions [barometric pressure (mmHg), air temperature (°c), and wind velocity (mph)], were used to assess the *F-V* relationship variables. These variables included the maximal theoretical horizontal force (*F_0_*, representing the horizontal force capacities at low velocities, in N∙kg^−1^), the theoretical maximal running velocity at which horizontal force could be generated (*V_0_*, representing the horizontal force capacities at high velocities, in m∙s^−1^), the slope of the *F-V* relationship (*S_F-V_*, in N∙s∙m^−1^∙kg^−1^), and the maximal propulsive power output (*P_max_*, in W∙kg^−1^). These measurements were computed using an Excel spreadsheet with a computational model validated as outlined in Morin et al. (2019).

### Statistical Analysis

Mean values and standard deviations were calculated for all examined variables. Normality and sphericity assumptions were assessed before employing parametric statistical tests. Normality was confirmed using histograms, the Kolmogorov-Smirnov and Shapiro-Wilk tests, while the Levene's test verified homoscedasticity.

A two-way analysis of variance (ANOVA) was conducted to explore potential interactions between dribbling and the playing position. Effect sizes for the two-way ANOVA were evaluated using partial eta squared (*η^2^*). In the event of an interaction effect (dribbling x playing position), a one-way ANOVA was conducted to assess differences between playing positions in outcomes changes (losses in %) between sprinting with and without a ball. Effect sizes for the one-way ANOVA were evaluated using partial eta squared (*η^2^*). Subsequently, post hoc tests utilizing the Tukey HSD method were employed to identify significant differences among the groups.

Effect sizes (*η^2^*) were interpreted as small (> 0.01), medium (> 0.06), or large (> 0.14) (Richardson, 2011). To assess the magnitude of differences for pairwise comparisons, Hedges' g was used for pairwise comparisons, with effect sizes (ES) of 0.2 considered small, 0.6 medium, 1.2 large, 2.0 very large, and 4.0 extremely large (Hopkins et al., 2009).

Pearson correlation coefficients (r) evaluated the relationships between *F-V* variables assessed during sprints with and without the ball, as well as between percentage losses in sprint performance (%losses -*T_5_*, -*T_20_*) and *F-V* profile losses (%losses -*P_max_*, -*F_0_*, -*V_0_*, and -*S_F-V_*). Qualitative interpretations of the correlation coefficient were as follows: trivial (r < 0.1), small (r = 0.1–0.3), moderate (r = 0.3–0.5), large (r = 0.5–0.7), very large (r = 0.7–0.9), and nearly perfect (r > 0.9) (Hopkins et al., 2009). All statistical analyses were conducted using SPSS software, version 23.0, with significance set at *p* ≤ 0.05.

## Results

### Dribbling and Position Effects on Performance

**Figure 1:**
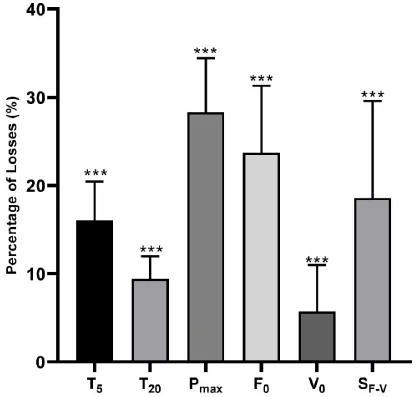
Percentage of losses in performance and the F-V profile for all players while dribbling compared to sprinting without the ball. ***: indicates a significant difference between sprinting with and without the ball at p < 0.001

Performance output across various sprints and playing positions is summarized in Table 1. The two-way ANOVA results indicated a significant main effect of dribbling on both *T_5_* and *T_20_* sprint performances when positions were pooled (*p* < 0.001; *η^2^*: 0.96, 0.94) (Figure 1).

Similarly, a significant main effect of the playing position was observed on *T_5_* and *T_20_* sprint performances when sprints were combined (*p* < 0.001; *η^2^*: 0.65, 0.74). Interactions between dribbling and playing positions were significant for *T_5_* (*p* < 0.001; *η*^2^ = 0.38) and *T_20_* (*p* = 0.003; *η*^2^ = 0.28). Post-hoc analysis in sprinting without the ball revealed higher *T_5_* for WMs and cMs compared to cDs (ES: 1.62, 1.25). Additionally, higher *T_20_* were observed for Fs (ES = 2.21), WMs (ES = 1.56) and cMs (ES = 1.34) compared to cDs. In dribbling sprints, WMs and Fs exhibited higher *T_5_* compared to cDs (ES: 1.8, 1.91), WDs (ES: 1.58, 1.63) and cMs (ES: 1.34, 1.36), with higher *T_20_* for Fs and WMs compared to cDs (ES: 2.76, 1.79), WDs (ES: 1.67, 1.57) and cMs (ES: 1.60, 1.48). Moreover, *T_20_* was lower for cDs compared to WDs (ES = 1.65) and cMs (ES = 1.22).

**Table 1:** Two-way ANOVA results for performance and F-V profile output across various sprints and positions. Values are presented as mean ± SD.

	Variables	Position	cD	WD	cM	WM	F	Dribbling Effect		Position Effect	Interaction Effect
Performance	*T_5_* (s)	Sprinting	1.30 ± 0.03	1.25 ± 0.04	1.24 ± 0.05^¶^	1.23 ± 0.04^¶^	1.25 ± 0.03	*p* < 0.001 *η^2^* = 0.96		*p* < 0.001 *η^2^* = 0.65	*p* < 0.001 *η^2^* = 0.38
		Dribbling	1.49 ± 0.05	1.49 ± 0.06	1.47 ± 0.06	1.40 ± 0.05^¶ ¥ €^	1.40 ± 0.04^¶ ¥ €^				
	*T_20_* (s)	Sprinting	3.37 ± 0.06	3.34 ± 0.08	3.27 ± 0.09^*^	3.24 ± 0.06^¶^	3.24 ± 0.11^¶^	*p* < 0.001 *η^2^* = 0.94		*p* < 0.001 *η^2^* = 0.74	*p* = 0.003 *η^2^* = 0.28
		Dribbling	3.78 ± 0.08	3.66 ± 0.06	3.68 ± 0.09	3.54 ± 0.09^¶ ¥ €^	3.52± 0.11^¶ ¥ €^				
Force-Velocity Profile	*F_0_* (N/kg)	Sprinting	8.93 ± 0.80	8.87 ± 0.64	10.02 ± 0.88^¶ ¥^	9.76 ± 0.67^¥^	9.63 ± 0.54	*p* < 0.001 *η^2^* = 0.91		*p* < 0.001 *η^2^* = 0.58	*p* = 0.02 *η^2^* = 0.22
		Dribbling	6.69 ± 0.67	6.74 ± 0.58	7.10 ± 0.80	7.80 ± 0.62^¶ ¥^	7.70 ± 0.66^¶ ¥^				
	*V_0_* (m/s)	Sprinting	8.29 ± 0.37	8.57 ± 0.25	8.34 ± 0.36	8.56 ± 0.26	8.68 ± 0.51	*p* < 0.001 *η^2^* = 0.56		*p* < 0.001 *η^2^* = 0.41	n. s. *p* = 0.62 *η^2^* = 0.05
		Dribbling	7.60 ± 0.18	8.20 ± 0.46	7.90 ± 0.46	8.05 ± 0.33	8.23 ± 0.35				
	*P_max_* (W/kg)	Sprinting	18.47 ± 1.35	19.01 ± 1.44	20.88 ± 1.82^¶ ¥^	20.89 ± 1.39^¶ ¥^	20.93 ± 1.67^¶ ¥^	*p* < 0.001 *η^2^* = 0.95		*p* < 0.001 *η^2^* = 0.75	*p* = 0.007 *η^2^* = 0.25
		Dribbling	12.69 ± 1.18	13.83 ± 0.94	13.94 ± 1.11	15.67 ± 1.16^¶ ¥ €^	15.84 ± 1.37^¶ ¥ €^				
	*S_F-V_* (N∙s∙m^−1^∙kg^−1^)	Sprinting	−1.08 ± 0.13	−1.04 ± 0.08	−1.21 ± 0.13	−1.14 ± 0.1	−1.12 ± 0.09	*p* < 0.001 *η^2^* = 0.72	*p* = 0.009 *η^2^* = 0.35		n. s. *p* = 0.16 *η^2^* = 0.14
		Dribbling	−0.88 ± 0.10	−0.83 ± 0.11	−0.91 ± 0.14	−0.97 ± 0.1	−0.95 ± 0.10				

cD: central defender; WD: wide defender; cM: central midfielder; WM: wide midfielder; F: forward; ¶: significantly different from cD; ¥: significantly different from WD; €: significantly different from cM; n. s.: non-significant interaction effect

**Figure 2:**
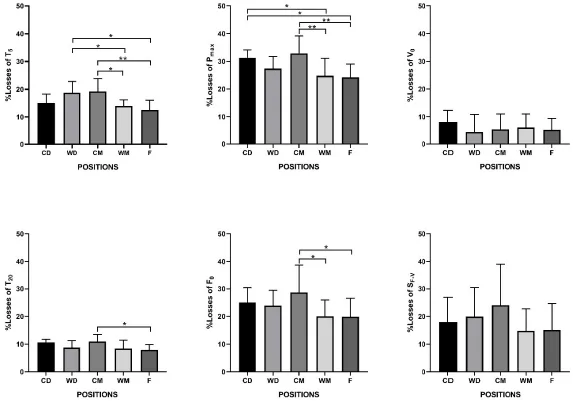
Percentage of losses of performance and the F-V profile for each playing position. cD: central defender; WD: wide defender; cM: central midfielder; WM: wide midfielder; F: forward; *: indicates a significant difference between positions with p < 0.05; **: indicates a significant difference between positions with p < 0.01

The one-way ANOVA demonstrated a significant effect of playing position for %losses-*T_5_* (*p* < 0.001; F_4, 47_ = 6.80; *η*^2^ = 0.37) and %losses-*T_20_* (*p* = 0.009; F_4, 47_ = 3.77; *η*^2^ = 0.25). Post hoc analysis indicated higher %losses-*T_5_* for cMs and WDs compared to Fs (ES: 1.62, 1.60) and to WMs (ES = 1.44). Additionally, a significantly higher %losses-*T_20_* for cMs compared to Fs (ES = 1.36) was observed (Figure 2).

### Dribbling and Position Effects on the F-V Profile

*F-V* profile output across various sprints and playing positions is summarized in Table 1. Two-way ANOVA results revealed a significant main effect of dribbling on all *F-V* variables for all players (*p* < 0.001; *η*^2^: [0.56–0.95]) (Figure 1). A significant main effect of the playing position was observed on *F-V* profiles when considering all sprints together (*p* < 0.01; *η*^2^: [0.35–0.75]). Significant interactions between dribbling and the playing position were found for *P_max_* (*p* = 0.007; *η*^2^ = 0.25) and *F_0_* (*p* = 0.02; *η*^2^ = 0.22), but not for *V_0_* (*p* = 0.62) and *S_F-V_* (*p* = 0.16). Post-hoc analysis in sprinting without the ball revealed higher *P_max_* for F, WM and cM players compared to cD (ES: 1.61, 1.79, 1.48) and WD (ES: 1.24, 1.33, 1.12) players. Additionally, higher *F_0_* was observed for cM compared to cD (ES = 1.28) and WD (ES = 1.45) players, and for WM compared to WD (ES =1.34) players. In dribbling sprints, higher *P_max_* for Fs and WMs compared to cDs (ES: 2.47, 2.54), WDs (ES: 1.75, 1.76) and cMs (ES: 1.54, 1.52) was observed, along with higher *F_0_* for F and WM compared to cD (ES: 1.57, 1.76) and WD (ES: 1.57, 1.75) players.

The one-way ANOVA demonstrated a significant effect of the playing position for %losses-*P_max_* (*p* < 0.001; F_4, 47_ = 5.97; *η*^2^ = 0.34), and %losses-*F_0_* (*p* = 0.03; F_4, 47_ = 3.03; *η*^2^ = 0.21). Post hoc analysis revealed significantly higher %losses-*P_max_* for cM compared to F (ES = 1.53) and WM (ES = 1.29) players. cMs also exhibited a higher %losses-*F_0_* compared to WMs (ES = 1.05) and Fs (ES = 1.01) (Figure 2).

### Relationships between F-V Profile Output in Sprints with and without the Ball

Moderate to large correlations were observed between sprint without the ball and dribbling sprint *F-V* profiles (Table 2). Specifically, a large correlation was found for *P_max_* (r = 0.66; *p* < 0.001), and *F_0_* (r = 0.50; *p* < 0.001). Additionally, a moderate correlation was noted for *V_0_* (r = 0.35; *p* < 0.01).

**Table 2:** Pearson correlation coefficients for relationships between the sprint and dribble F-V profile output.

		Sprint without the ball		
	Variable	P_max_(W/)	F_0_ (N/kg)	V_0_ (m/s)
Dribbling sprint	DB-P_max_ (W/kg)	0.66***	0.51***	0.32*
	DB-F_0_ (N/kg)	0.57***	0.50***	0.16
	DB-V_0_ (m/s)	0.23	0.06	0.35**

Significant correlations: (*: p < 0.05; **: p < 0.01; ***: p < 0.001)

### Relationships between Loss in Performance and Loss in F-V Profiles

**Figure 3:**
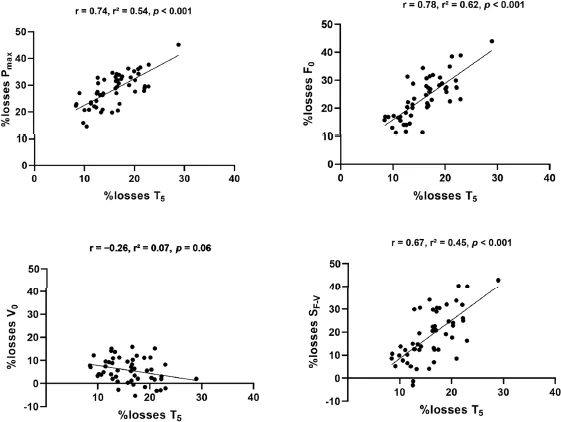
Correlation between the percentage of losses in sprint performance at the 5^th^ m and the percentage of losses in all F-V profiles.

**Figure 4:**
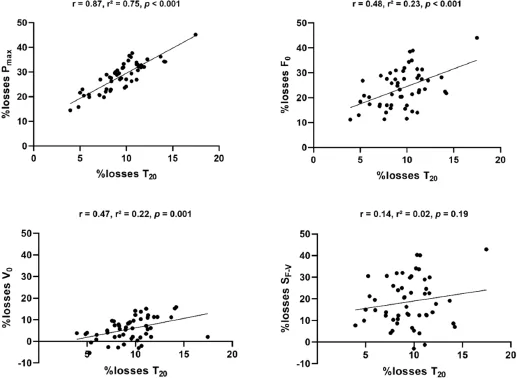
Correlation between the percentage of losses in sprint performance at the 20^th^ m and the percentage of losses in all F-V profiles.

Large to very large positive correlations were observed between %losses-*T_5_* and %losses-*P_max_* (r = 0.74; *p* < 0.001), %losses-*F_0_* (r = 0.78; *p* < 0.001), and %losses-*S_F-V_* (r = 0.67; *p* < 0.001). Notably, no correlation was found for %losses-*V_0_* (r = −0.26; *p* = 0.06) (Figure 3). Regarding the percentage of losses in *T_20_*, a substantial positive correlation was identified for %losses-*P_max_* (r = 0.87; *p* < 0.001). In addition, moderate correlations were found between %losses-*T_20_* and %losses-*F_0_* (r = 0.48; *p* < 0.001) and %losses-*V_0_* (r = 0.47; *p* = 0.001). However, no correlation was revealed for %losses-*S_F-V_* (r = 0.14; *p* = 0.19) (Figure 4).

## Discussion

This study aimed to assess the impact of dribbling on sprint performance and horizontal force production capacities in professional senior soccer players across various playing positions. Findings showed a clear decline in sprint performance and changes in the *F-V* profile while dribbling, notably at lower speeds. conversely, this effect was less pronounced at higher speeds, where players covered longer distances with the ball. These results highlight the intricate relationship between dribbling and sprinting dynamics, emphasizing the crucial role of acceleration and horizontal force production, especially at lower velocities, in sustaining performance during dribbling sprints.

As expected, incorporating dribbling led to a decline in sprint acceleration performance, with an average loss of 16% for *T_5_*, and 10% for *T_20_* (all position pooled). These findings align with previous studies reporting sprint split-time reductions ranging from 10 to 30% for soccer players when dribbling (Lupo et al., 2019; Manouras et al., 2023). Notably, variations in the impact of dribbling on sprint performance were observed among playing positions at both *T_5_* and *T_20_*. cM and WD players exhibited a substantial decline in *T_5_* compared to F and WM players, with cMs experiencing an even greater decline in *T_20_* compared to Fs. These variations were likely due to documented differences in physical capacity and technical ability when in possession of the ball (Altmann et al., 2021; Dellal et al., 2011; Di Salvo et al., 2007; Vigh-Larsen et al., 2018). Studies indicate that F and WM players engage in more high-intensity activities (Altmann et al., 2021; Vigh-Larsen et al., 2018), with WMs covering greater distances while possessing the ball compared to cM and cD players (carling, 2010; Dellal et al., 2011; Di Salvo et al., 2007). These differences suggest higher physical sprinting abilities among F and WM players, highlighting their impact on technical skills.

Our results reveal varied changes in the *F-V* profile between sprints with and without the ball across playing positions, particularly in *P_max_* and *F_0_*. cMs and cDs exhibited greater *P_max_* loss than WMs and Fs, with cMs exhibiting higher *F_0_* loss than WMs and Fs. Despite position-specific variations in the *F-V* profile, the loss in *V_0_* remained consistent across positions, indicating a uniform effect of dribbling on horizontal force production at high velocities. These position-specific variations underscore the need for tailored training programs to optimize performance during dribbling situations across different playing positions. Additionally, individualized strategies may be necessary to mitigate the negative effects of dribbling on horizontal force production, particularly when players need to accelerate during gameplay.

Notably, the magnitude of loss in the *F-V* profile was higher for *F_0_* (24%) and *P_max_* (28%) compared to *V_0_* (6%), highlighting the challenge of dribbling while generating substantial horizontal force, especially at low (without propelling the ball too far due to reduced speed), and less so at higher velocity (where longer dribbles facilitated the dual task). This controlled motion, crucial for effective ball control, inherently limits athletes' capacity for maximal sprinting efforts. These insights into *F-V* profile variations between sprints without the ball and dribbling sprints across playing positions offer novel perspectives in soccer literature, enabling coaches to tailor interventions for optimized player development.

Results illustrate reduced horizontal force production capacities while dribbling compared to sprinting without the ball, hinting at potential technical skill deficits, notably observed in cMs. cDs displayed greater loss in *P_max_* compared to offensive positions, while WDs showed no significant differences despite sprint performance variations. These findings underscore the complex relationship among playing positions, dribbling, and sprinting mechanics, emphasizing the necessity for a nuanced comprehension of these interactions.

Fs and WMs demonstrated higher *P_max_* and *F_0_* during sprints without the ball, with the lowest loss in *P_max_* and *F_0_*, suggesting superior sprint acceleration performance while dribbling compared to other positions. Offensive players, due to their specific roles, demonstrate greater familiarity with executing sprint actions with and without the ball (carling, 2010) and exhibit superior dribbling technique (Bradley et al., 2011). Previous studies have also shown that WMs cover the greatest distance, spend the most time in possession, and have a higher number of touches per possession (carling, 2010; Dellal et al., 2011). Additionally, Fs and WMs achieve the highest speed in possession, outperforming cDs and cMs (carling, 2010; Oliva-Lozano et al., 2023).

cDs showed lower *P_max_* and *F_0_* during sprints without the ball, with the most significant changes in the sprinting *F-V* profile observed during dribbling, compared to Fs and WMs. Variations could be attributed to differences in sprinting and dribbling distances and occurrences, influenced by the technical and tactical roles of particular positions (Dellal et al., 2011). Specifically, cDs focus more on preventing goals, winning duels, and intercepting the ball from opponents (Dellal et al., 2011).

cMs displayed greater changes in the sprinting *F-V* profile while dribbling compared to Fs and WMs, despite similar performances in sprints without the ball. This highlights a specific challenge for cMs, indicating difficulty in maintaining maximal sprinting performance while dribbling. Existing literature suggests that cMs exhibit lower maximum speeds during match play than other positions (Oliva-Lozano et al., 2023). Additionally, cMs engage in fewer sprints per match (≈6) compared to Fs and WMs (≈16) (Andrzejewski et al., 2013; Oliva-Lozano et al., 2020). Their primary responsibilities involve successful passes, defending, and attempting to win possession in the middle of the pitch. Unlike offensive positions (WMs, Fs), the technical demands associated with sprinting may not be as critical for central players (cDs, cMs).

Correlation analyses revealed significant relationships between sprint without the ball and dribbling sprint *F-V* profiles, emphasizing their shared characteristics regardless of ball possession. These findings suggest that *F-V* profiles during sprints without the ball influence performance in dribbling sprints. Furthermore, correlations between sprint performance decline and *F-V* profile changes underscore the impact of maximal power loss on dribbling sprint performance. Decreased force production capacity at low velocity correlated with decline in 5-m split time, while decline in both low and high velocities correlated with decline in 20-m split time. This highlights the need to optimize this key *F-V* output for sustained high-level performance in dynamic athletic contexts.

This study unveils position-specific variations in the *F-V* profile during dribbling, laying the groundwork for future biomechanical investigations in soccer players. Analyzing dribbling kinematics could elucidate the interplay between sprinting mechanics and ball handling skills. The decreased force production capacity observed in our study could be attributed to players having less time to apply force while dribbling, thereby reducing their ability to generate impulse and effectively accelerate. closely examining the stride alterations induced by dribbling and their impact on force output will offer valuable insights for coaches and trainers. Understanding position-specific demands informs targeted training interventions to optimize force production during sprinting and enhance on-field dribbling proficiency for all players.

## Study Limitations

While providing valuable insights, our study has some limitations which should be acknowledge. A primary limitation is the reliance on video-based analysis instead of advanced technologies such as the GPS, radar, linear encoders or laser systems. These devices are widely regarded as more precise for capturing sprint mechanics and *F-V* profiles. Their ability to measure variables such as foot contact time and in-game movements with high temporal and spatial resolution makes them superior tools for analyzing performance in realistic, in-match scenarios. Future research should prioritize incorporating these advanced technologies to enhance data accuracy and provide a deeper understanding of athletes' performances under competitive conditions.

Additionally, there was a slight time discrepancy in our study between the defined initiation of the sprint (observable forward movement) and the exact moment when the subject began producing force on the ground (heel sinking). This methodological decision, while simplifying detection and minimizing random errors, may have caused slight overestimation of *F_0_* values. However, we are confident that this does not introduce systematic bias, as it does not affect the validity of comparisons across sprint conditions or playing positions.

Finally, our study focused exclusively on linear sprint performance. Future investigations should consider incorporating various dribbling tests, such as change of direction and agility performance, to provide a more comprehensive understanding of soccer players' technical and physical abilities.

## Practical Implications

This study offers important insights into how dribbling affects sprint performance and horizontal force production, highlighting the unique challenges faced by players in different positions. Based on our findings, soccer training programs should be tailored to account for the specific challenges players face while dribbling, especially regarding force production at lower velocities. For all players, incorporating short-distance dribbling sprints (5 to 10 m) into training is essential to enhance sprint acceleration while dribbling the ball. These exercises help improve the ability to generate horizontal force during initial acceleration, which is critical for maintaining performance when dribbling.

When considering the playing position, our results showed that offensive players, such as forwards and wide midfielders, experienced minimal loss in sprint *F-V* profiles while dribbling. Their higher sprinting capacities and better dribbling techniques made them more adept at combining sprinting and ball control. As a result, for these players, training should prioritize maintaining or enhancing their sprinting performance while continuing to integrate dribbling drills. conversely, for central midfielders, who experienced more significant reductions in sprint *F-V* profiles during dribbling, specific targeted training is necessary. These players often struggle to maintain maximal sprinting performance while dribbling due to their lower *F-V* profiles during sprints without the ball. Therefore, their training should initially prioritize improving basic sprinting abilities, focusing on force production at lower velocities, before introducing ball-related drills.

Incorporating these position-specific insights into training programs will allow coaches to effectively address the unique needs of individual players, enhancing their performance in both sprinting and dribbling contexts. By conducting detailed sprint analyses with and without the ball, coaches can identify players who experience notable performance decline during dribbling. This process allows for customized training programs designed to address specific weaknesses while promoting targeted performance improvements. Adopting a two-phased approach—initially focusing on enhancing sprint mechanics and subsequently integrating ball control into training—can further optimize the combined benefits of these elements, ultimately enhancing players' on-field effectiveness.

## conclusions

In summary, our study confirms the impact of dribbling constraints on sprint acceleration performance and the *F-V* profile across playing positions. We observed a distinct decrease in the *F-V* profile during dribbling sprints among positions, with more pronounced alterations in *P_max_* and *F_0_* compared to *V_0_*. The decline in sprint acceleration performance during dribbling could be primarily attributed to a decrease in *F_0_* rather than *V_0_*. These findings underscore the importance of tailoring training to specific positions, considering both sprinting and dribbling *F-V* profiles. coaches can optimize players’ development by understanding these factors and designing customized training programs for professional soccer players.
